# Binder-Free Cathode for Thermal Batteries Fabricated Using FeS_2_ Treated Metal Foam

**DOI:** 10.3389/fchem.2019.00904

**Published:** 2020-01-10

**Authors:** In Yea Kim, Sung Pil Woo, Jaehwan Ko, Seung-Ho Kang, Young Soo Yoon, Hae-Won Cheong, Jae-Hong Lim

**Affiliations:** ^1^Department of Materials Science and Engineering, Gachon University, Seongnam, South Korea; ^2^Department of Materials Science and Engineering, Yonsei University, Seoul, South Korea; ^3^Agency for Defense Development, Daejeon, South Korea

**Keywords:** thermal battery, FeS_2_ foam, metal foam, thermal sulfidation, cathode frame

## Abstract

In this study, we fabricated a cathode with lower amounts of additive materials and higher amounts of active materials than those of a conventional cathode. A thermal battery was fabricated using FeS_2_ treated foam as the cathode frame, and its feasibility was verified. X-ray diffraction, transmission electron microscopy, and scanning electron microscopy were used to analyze the effects of thermal sulfidation temperature (400 and 500°C) on the structure and surface morphology of the FeS_2_ foam. The optimal temperature for the fabrication of the FeS_x_ treated foam was determined to be 500°C. The FeS_2_ treated foam reduced the interfacial resistance and improved the mechanical strength of the cathode. The discharge capacity of the thermal battery using the FeS_2_ treated foam was about 1.3 times higher than that of a thermal battery using pure Fe metal foam.

## Introduction

Thermal batteries are potential power sources for nuclear weapons and warhead missiles and are used in aerospace applications owing to their excellent mechanical properties, reliability, and low self-discharge characteristics (Guidotti and Masset, 2006; Kang et al., 2016; Jin et al., 2018). Cathodes of commercial thermal batteries are fabricated using transition metals such as FeS_2_, CoS_2_, and NiS_2_; their anodes are fabricated with Li-alloys (Li-Si and Li-Al); and a eutectic mixture of LiCl-KCl, which exists in the molten state at the operating temperature of thermal batteries (~500°C), is used as the electrolyte (Guidotti and Masset, 2008; Masset and Guidotti, 2008a; Giagloglou et al., 2016). Although the electrochemical properties of CoS_2_ and NiS_2_ are similar to those of FeS_2_, sulfur exhibits a high loss rate when it encounters electrolytic salts (Preto et al., 1983). In addition, synthesis of CoS_2_ is expensive, thereby limiting its usage in large-scale applications (Jin et al., 2017).

Generally, cathodes of thermal batteries are in the form of cold-pressed pellets. To manufacture a pellet-type cathode, the cathode active material is mixed with various additives such as salts, electrolyte, and a binder (Au, 2003; Singh et al., 2004; Masset et al., 2005; Yang et al., 2014). Such cathodes are called catholytes (cathode + electrolyte) because they contain a large amount of electrolyte to transfer ions to the cathode during the operation of thermal batteries (Au, 2003; Guidotti and Preston, 2007; Masset and Guidotti, 2008a; Yang et al., 2014). Moreover, binder materials such as MgO and SiO_2_ are added to the cathode to ensure the molten salt is maintained at the high operating temperatures of thermal batteries (Kim et al., 2017; Cha et al., 2018; Wu et al., 2018). However, these additives increase the internal resistance of the electrodes, and their addition reduces the amount of cathode active material, thus deteriorating the discharge characteristics of the cathode (Masset and Guidotti, 2007; Ko et al., 2017). In addition, it is difficult to manufacture large-sized cathodes with sufficient mechanical strength through a cold-press method (Guidotti et al., 2000, 2002; Leviatan, 2011).

Various methods to manufacture reliable cathodes with sufficient mechanical strength have been studied. The most notable methods are tape casting and thermal spraying. Via tape casting, a cathode can directly be fabricated onto the current collector or on a graphite paper substrate from slurry by blending the active cathode material and some polymeric binders, thus fabricating thin cathodes; the size of the cathodes can be easily scaled up. However, the process of manufacturing the slurry is complicated, and the method increases the internal resistance of the batteries because of the presence of various polymer binders (Masset et al., 2005; Cha et al., 2018). Thermal spraying is a coating process in which the FeS_2_ active material is sprayed through a heated feedstock onto the surface of a prepared current collector. In this method, the surface of the current collector is coated by spraying raw materials. Electrode formation by thermal spraying is simpler than that by tape casting. However, decomposition of FeS_2_ may lead to the deposition of FeS or S_2_ in the heated feedstock, causing a spike during thermal battery discharge (Guidotti and Preston, 2007). In addition, the thermal spray method requires expensive equipment. However, electrodes fabricated by the two methods mentioned are very thin to allow loading of large active materials. A small amount of active material in the positive electrode degrades the discharge characteristics of thermal batteries. Therefore, a simple, inexpensive method for fabricating cathodes containing the necessary active materials with lesser additives needs to be developed.

**Figure 1:**
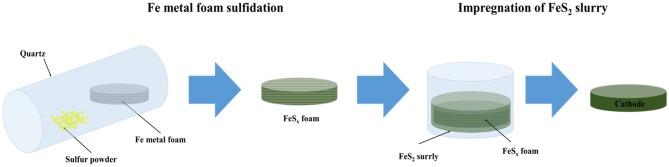
Schematic representation of the synthesis process of the cathode using FeS_x_ foam for thermal batteries.

Thermal battery cathodes have been fabricated using metal foam as an electrode support and conductor (Ji et al., 2012; Sun et al., 2019). Metal foams of various sizes and thicknesses with high porosity (>90%) can be added to various active materials to improve mechanical strength. However, metal foams increase the contact resistance with the active material because of low structural similarity with the cathode active material. Furthermore, they reduce the capacity of the batteries because the metal does not participate in the electrochemical reaction during thermal battery operation. To improve these characteristics, the metal foam needs to be treated with a material with structural characteristics similar to those of the active material of the cathode. Fe metal foam is a promising option because it can react with S_2_ powders to form FeS_2_, a thermal battery cathode material, via simple thermal sulfidation, as shown in Figure 1 (Ferrer and Sánchez, 1991; Liu et al., 2016). The purpose of this study is to synthesize a cathode frame whose structural characteristics are the most similar to those of cathode materials (FeS_2_) of thermal batteries. In addition, the thermal battery discharge characteristics were evaluated. The discharge performance of a thermal cathode with FeS_2_ foam and that with Fe foam in a single cell configuration were compared to evaluate the efficiency of the sulfidation reaction. The discharge capacity of the unit cell with the synthesized FeS_2_ foam electrode was 538.3 A s/g at the cut-off value of 1.3 V and that for the single cell with Fe metal foam was 404.04 A s/g. These results indicate that the use of FeS_2_ foam is an effective method to fabricate thermal battery cathodes.

## Experimental

Thermal sulfidation of FeS_2_ foam was carried out in a sealed quartz tube to prevent oxidation and incorporate the evaporated sulfur. Fe foam (porosity: 95%, pore size: ~450 μm, Alantum Corporation, Seongnam, South Korea) and sulfur powder (purity: 99%, Sigma Aldrich, St. Louis, Missouri, USA) were used as starting materials to fabricate the FeS_2_ foam. The Fe foam was cut into a circle of diameter 56.2 mm, which was the size of the cathode. The Fe foam and sulfur powder (twice the amount of Fe in terms of molar ratio) were placed in the quartz tube, which was purged with Ar gas and subsequently sealed. The precursors placed in the quartz tube were then sulfidized at 400 and 500°C (HT-400 and HT-500) for 3 h. The heating rate was maintained at 2°C/min. After thermal sulfidation, the quartz tube was slowly cooled to room temperature. All the experiments were performed in a glove box to prevent contact with oxygen and moisture.

FeS_2_ slurry was impregnated into FeS_2_ foam to fabricate the thermal battery cathodes. The FeS_2_ slurry was prepared as follows. FeS_2_ powder (mean size: 98 μm, purity: 99% LinYi, China) was dispersed in acetone by ball milling using a BYK-111 (Altana) dispersant for 24 h. The initial particle size of the FeS_2_ powder was about 98 μm; however, the particle size reduced to about 20 μm after ball milling for 24 h. The size reduction of the particles by ball milling has been confirmed in previous studies (Ko et al., 2017). The FeS_2_ slurry was placed in a glass petri dish along with Fe and FeS_2_ foam, and the resulting mixtures were left under ambient conditions for several minutes. The mixtures were subsequently dried at 70°C for 24 h to completely remove acetone. A schematic representation of the entire process of cathode fabrication via FeS_2_ impregnation on Fe foam and the FeS_2_ foam prepared by thermal sulfidation is shown in Figure 1.

The crystalline phases of FeS_2_ foams, which were synthesized at different sulfidation temperatures, were analyzed by X-ray diffraction (XRD, PANalytical X'Pert PRO) with Cu Kα radiation. The surface morphological properties and element ratios of the samples were examined using a field-emission scanning electron microscope (Hitachi S-4200) with an energy dispersive X-ray spectrometer (EDX). The resistances of the FeS_2_ foams were measured at room temperature by electrochemical impedance spectroscopy (EIS, IM6Ex) over a frequency range of 100 mHz−2 MHz with an amplitude voltage of 10 mV.

**Figure 2:**
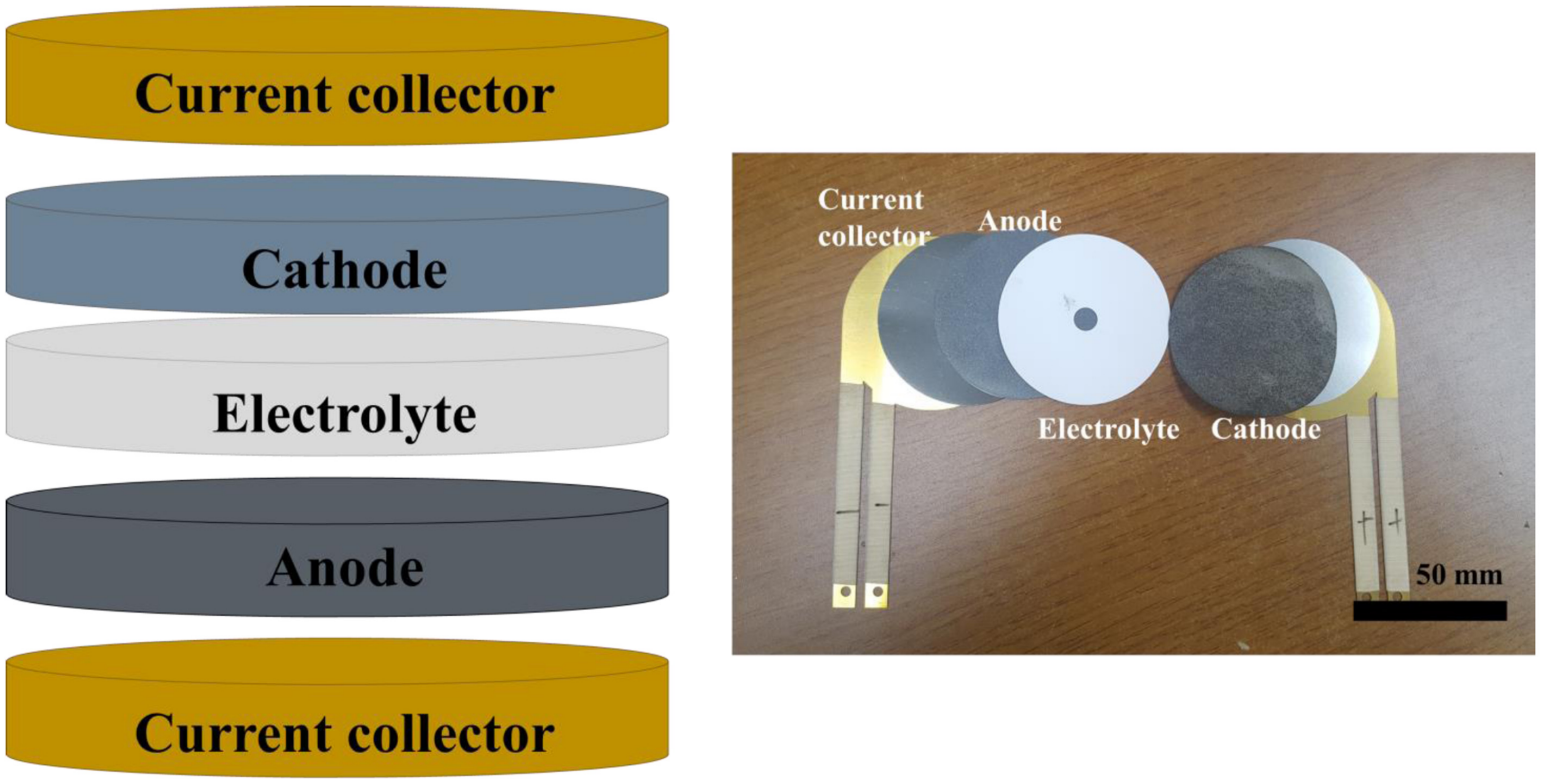
Structure and images of a single cell for a thermal battery.

The cathodes fabricated using the pure Fe and FeS_2_ foams were used to evaluate the discharge characteristics of thermal battery single cells. Figure 2 shows the general procedure of assembly of the single cell used to confirm the discharge characteristics of the cathode using HT-500 foam.

To verify the characteristics of the cathode, a commercial pellet-type electrode was used as the separator and anode. The discharge test of the single cell was performed under conditions similar to the operating conditions of thermal batteries and by using Daque 9,000 at 500°C with a load of 250 kg_f_ while applying a consecutive pulse current (10 A, 4.5 s → 0 A, 0.5 s). The discharge test was terminated when the voltage dropped below 1.3 V.

## Results and Discussion

### FeS_2_ Foam Fabrication According to Sulfidation Temperature

**Figure 3:**
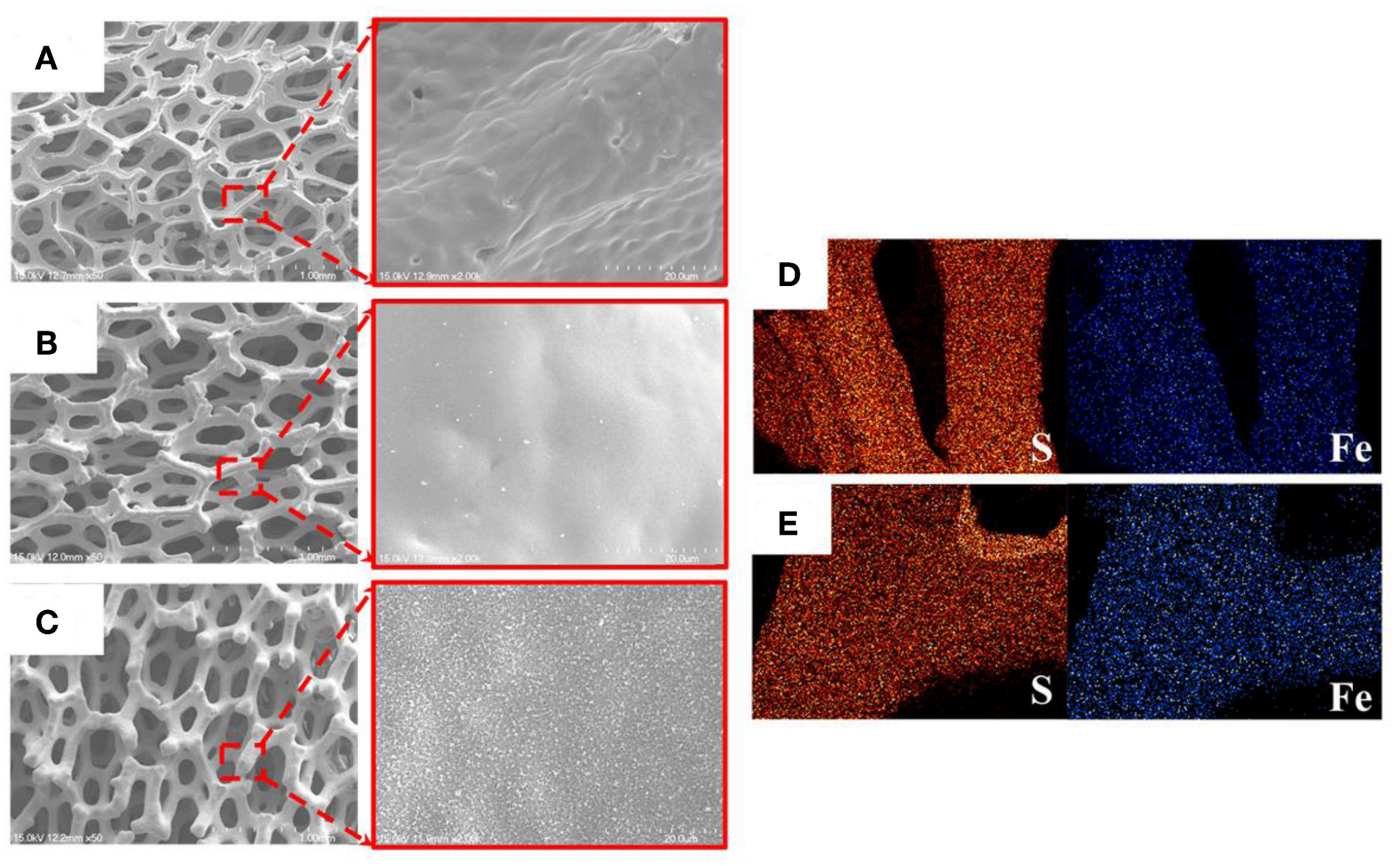
SEM images of **(A)** Fe, **(B)** HT-400, and **(C)** HT-500 foams. EDX images of **(D)** HT-400 and **(E)** HT-500 foams.

FeS_2_ foam was fabricated via thermal sulfidation at 400 and 500°C on Fe foams. The surface morphologies of the Fe and FeS_2_ foams synthesized at different sulfidation temperatures were examined by SEM. Figure 3A shows that the Fe foam had a three-dimensional structure with interconnecting holes (200–450 μm) made up of arch ribs. The arch ribs of the Fe foam had smooth and uniform wrinkles on the highly porous surface. Figures 3B,C shows that HT-400 and HT-500 foams were prepared at different thermal sulfidation temperatures. SEM images of the foams treated by thermal sulfidization revealed that sulfur was deposited entirely on the surface of the arch ribs. Both HT-400 and HT-500 foams had porous structures with no wrinkles on their surfaces (Figures 3B,C) unlike the Fe foam. As the thermal sulfidation temperature increased, the amount of sulfur deposited on the surface of the arch ribs increased along with an increase in the surface grain size. However, the synthesized HT-400 and HT-500 foams increased in terms of grain size with increasing sulfidation temperature but did not exhibit pores and cracks (Figure S1). These results show an improvement in surface conditions over those obtained by Rahman and Wen (2016). In addition, the synthesized HT-400 and HT-500 foams are expected to exhibit stable mechanical strength because they have 3D architectures. Moreover, EDS mapping clearly showed the uniform distribution of S and Fe on the surface of the foams (Figures 3D,E). HT-500 foam consisted of 32.79% of Fe and 67.21% of S with an Fe/S ratio of 2.04, which is very close to the atomic ratio in the formula FeS_2_ (Table 1). However, HT-400 foam consisted of 38.29% of Fe and 61.17% of S with an Fe/S ratio of 1.60. In other words, the sulfur content of the HT-400 foam was lesser than that of FeS_2_ (the material of thermal battery cathodes).

**Table 1:** Quantification results of EDX at different annealing temperatures.

**Heat treatment temp. (^**°**^C)**	**Concentration (at %)**	
	**Fe**	**S**
400	38.29	61.17
500	32.79	67.21

**Figure 4:**
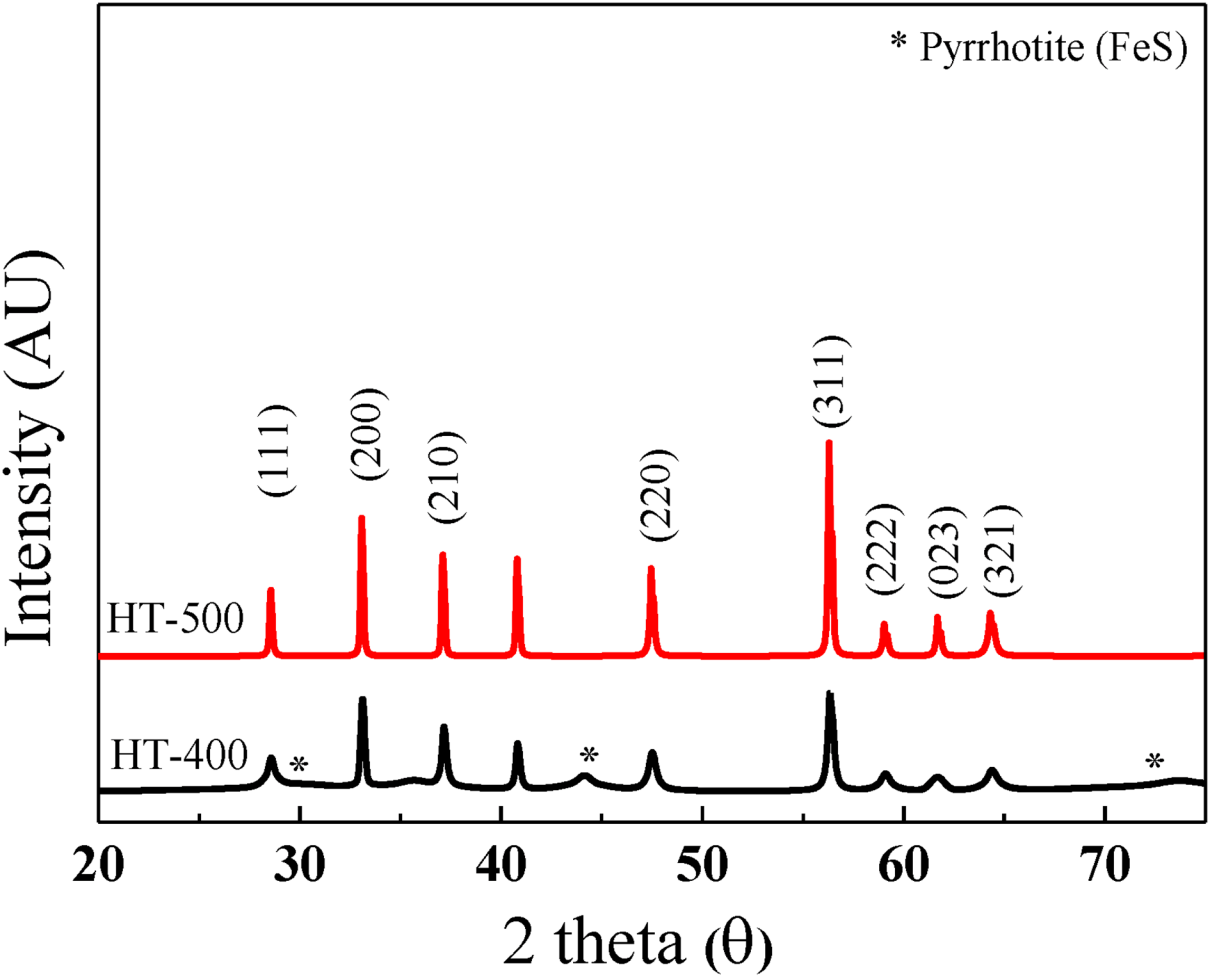
XRD patterns of HT-400 and HT-500 foams.

Figure 4 shows the XRD peaks of HT-400 and HT-500 foams. Both samples showed peaks corresponding to the (111), (200), (210), (211), (220), (311), (222), (023), and (321) planes (JCPDS card no. 42-1340). This exhibits a cubic pyrite (FeS_2_) structure with the space group of *Pa3* (Cabán-Acevedo et al., 2013; Lucas et al., 2013; Miao et al., 2017). However, XRD peaks for FeS of HT-400 foam and the high Fe content by EDS analysis (in Table 1) indicate the coexistence of FeS and FeS_2_. Pyrrhotite (FeS) causes thermal battery degradation owing to self-discharge by pyrolysis and impurity formation during the operation of thermal batteries (Schoeffert, 2005; Masset and Guidotti, 2008b). In contrast, the HT-500 foam confirmed good crystallinity with high intensity and sharp XRD peaks. To further analyze the crystallinity and phase purity of HT-400 and HT-500 foams, HRTEM and SAED analyses were carried out on them (Figure S2). The HT-400 foam showed lattice spacings of 3.126 and 2.050 Å corresponding to the pyrite (111) and pyrrhotite (208) planes, respectively (Figure S1b). However, the HT-500 foam showed a lattice spacing of 3.126 Å corresponding to the (111) plane (Figure S1c).Thus, the structure of HT-500 foam was similar to that of the cathode material, and HT-500 foam did not contain any FeS; it was expected that there would be no incidental reaction related to FeS during thermal battery discharge.

**Figure 5:**
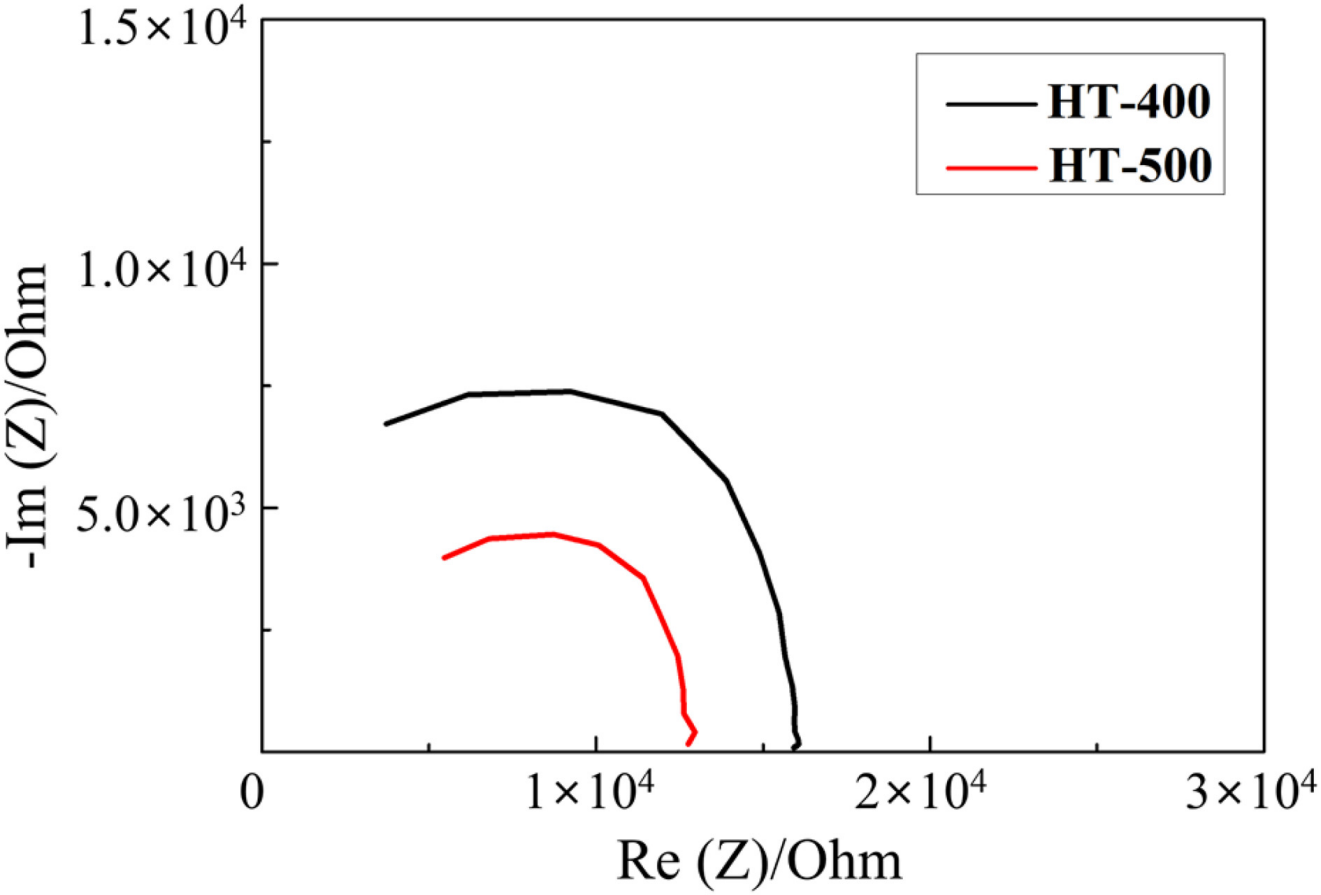
Nyquist plots of HT-400 and HT-500 foams.

Figure 5 shows the Nyquist plots of the HT-400 and HT-500 foams obtained from their EIS results (to evaluate their electrochemical properties). From the EIS results, the total ohmic resistance associated with the series resistance of HT-400 and HT-500 foams was determined. The high-frequency intercept at the real axis (Z) corresponds to the total ohmic resistance (R_s_) (Cooper and Smith, 2006; Gomez et al., 2011). In addition, a Warburg tail was not observed at low frequencies, indicating that only electrical conduction occurred (without ion conduction) at these frequencies (Sinclair, 1995). The electrical resistances of the HT-400 and HT-500 foams at room temperature were found to be 1.55 × 10^4^ and 9.37 × 10^3^ Ω, respectively. The diameter of the Nyquist curve semicircle of the HT-400 foam was larger than that of the HT-500 foam, indicating that the electrical resistance of the HT-400 foam was higher than that of the HT-500 foam.

These results suggest that the HT-400 foam was synthesized with a mixed phase rather than a single phase, thereby resulting in an increase in the total resistance (Thomas et al., 1998). The structure of HT-500 foam was similar to that of FeS_2_, and HT-500 foam exhibited more stable electrochemical properties than HT-400 foam. Therefore, HT-400 foam is not discussed in detail in the rest of the study.

### Single Cell Assembly Using Synthesized FeS_2_ Foam

**Figure 6:**
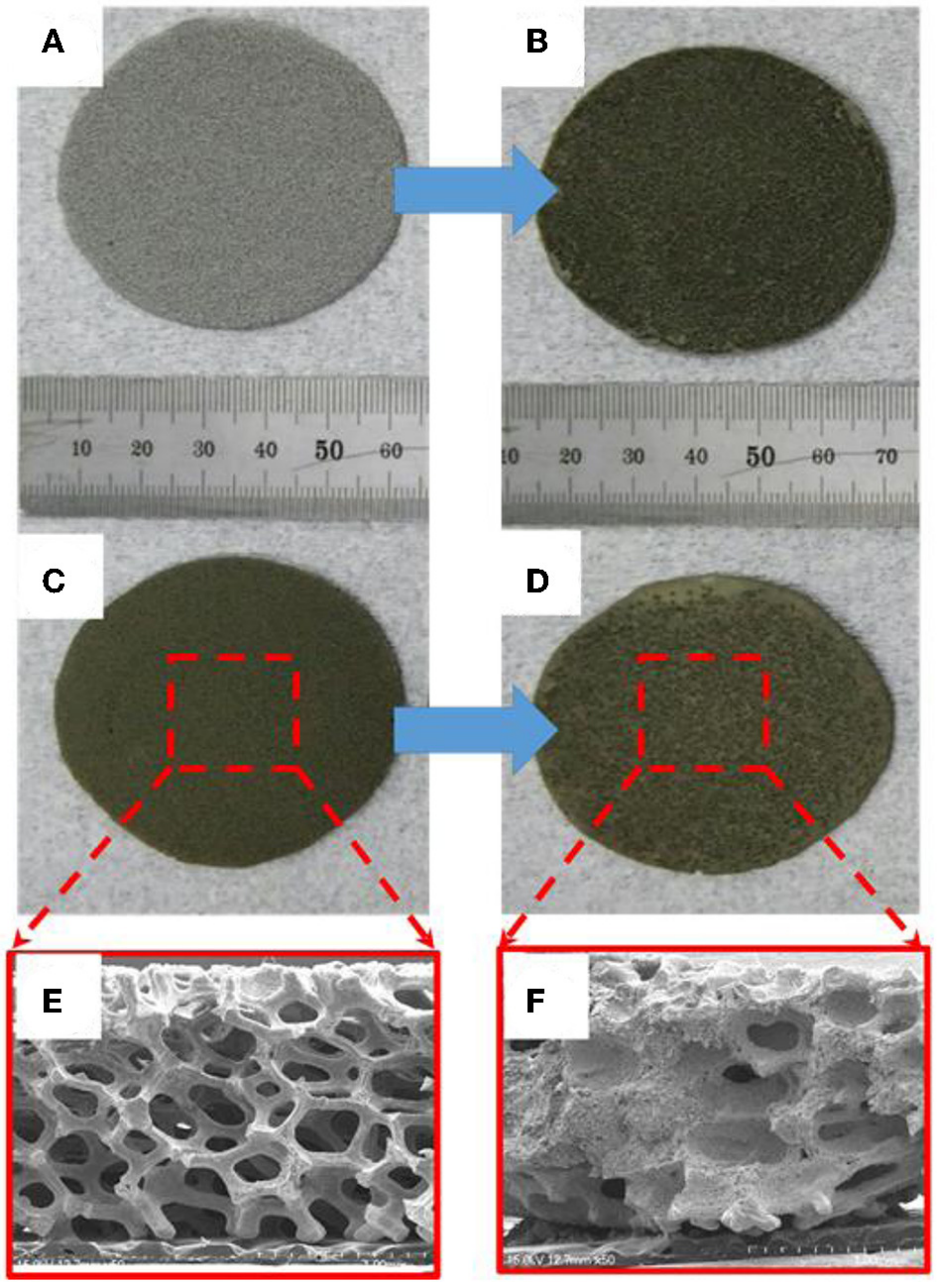
Image of pure Fe foam **(A)** before and **(B)** after impregnation with FeS_2_ slurry. HT-500 foam **(C)** before and **(D)** after impregnation with FeS_2_ slurry. Cross-section of the SEM image of HT-500 **(E)** before and **(F)** after impregnation with FeS_2_ slurry.

Cathodes for thermal batteries were fabricated using HT-500 and Fe foams to confirm the effect of thermal sulfidation. Both the cells were 16-mm thick and had a diameter of 56.2 mm. Figures 6A,B show images of the Fe foam before and after immersion in FeS_2_ slurry, respectively. The color of the Fe foam changed after immersion in the FeS_2_ slurry, indicating that the Fe foam was well-impregnated with the slurry. Figures 6C,D show images of the HT-500 foam before and after FeS_2_ slurry impregnation, respectively. The color of the FeS_2_ slurry impregnated HT-500 foam was similar to that of the FeS_2_ slurry impregnated Fe foam. Figures 6E,F show cross-sectional SEM images of the HT-500 foam before and after FeS_2_ slurry impregnation, respectively. The figures show that before impregnation, the HT-500 foam comprised void spaces. Such a frame structure can prevent separation of the cathode powder at the operating temperature of thermal batteries and can contain a large amount of active material. After impregnation, a large amount of fine FeS_2_ particles filled the voids and increased the density of the HT-500 foam.

### Evaluation of Discharge Characteristics of the Assembled Single Cells

**Figure 7:**
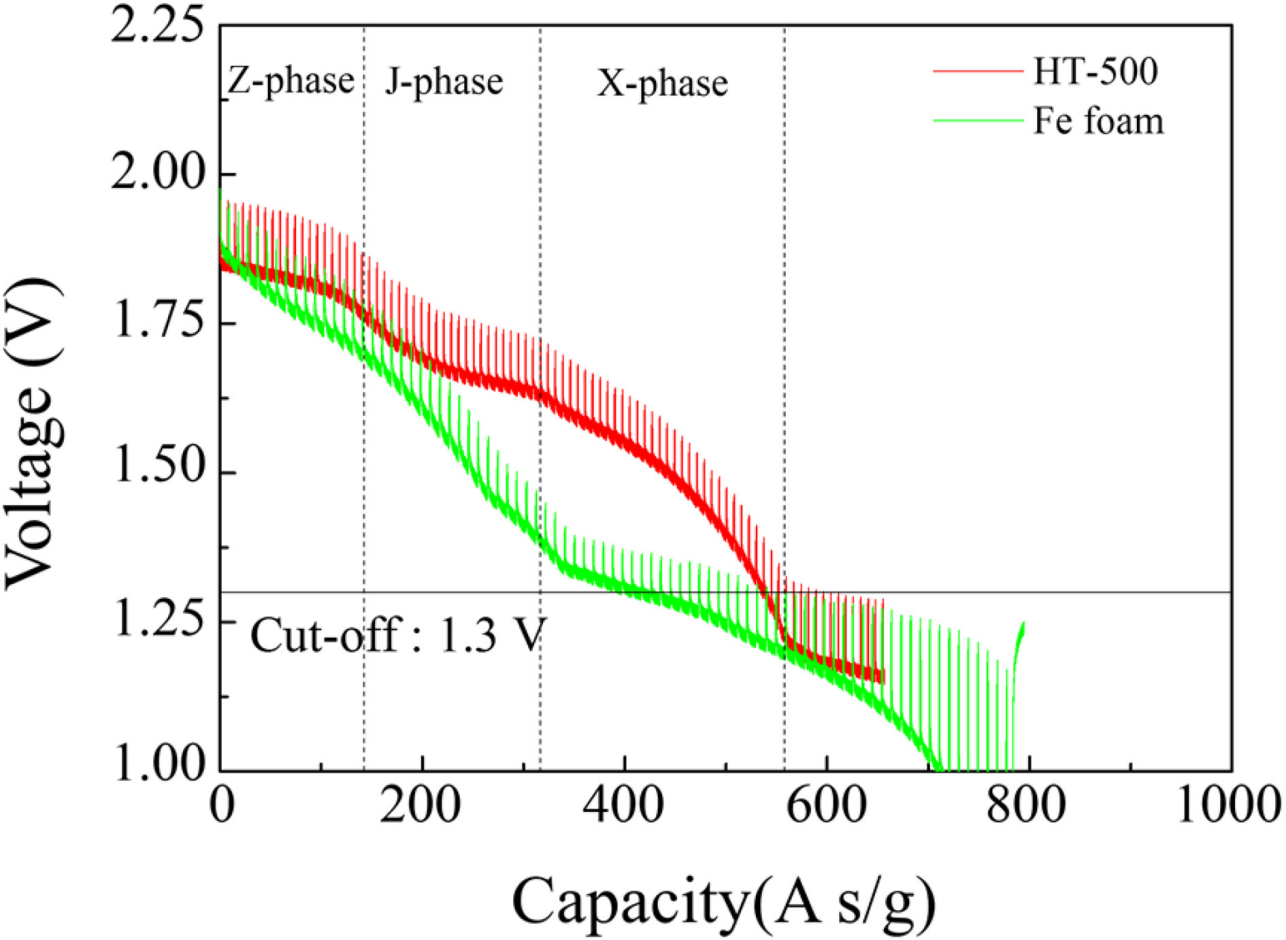
Discharge graphs of cathodes using Fe and HT-500 foams.

Figure 7 shows the discharge performance of the HT-500 and Fe foam cathodes and the assembled cells. The fabricated cells were discharged into the pulse mode at the thermal battery operating temperature. The inversion voltage plateaus of the thermal battery using FeS_2_ had three parts: the Z-phase reacts with FeS_2_ and Li to form Li_2_Fe_2_S_4_, the J-phase generates Li_3_Fe_2_S_4_, and the X-phase was the final reaction step to produce elemental lithium sulfur and iron (Choi et al., 2014). The X-phase occurs at 1.3 V (Ulissi et al., 2018).

In this study, we confirmed the capacity of the single thermal cell by setting the cut-off value to 1.3 V, which is the voltage at which the main reaction of the thermal battery cathode is completed, to confirm the final reaction of FeS_2_. Table 2 lists the thicknesses, foam weights, total electrode weights, and discharge capacities of the cathode using Fe and HT-500 foams. To determine the effect of Fe and HT-500 foams, the thermal battery capacity was calculated by considering the total cathode weight. The discharge capacity of the Fe foam cathode at the cut-off value of 1.3 V was found to be 404.04 A s/g. In contrast, the cell using the HT-500 foam cathode showed a discharge capacity of 538.38 A s/g. The reason for the higher discharge capacity of the HT-500 foam was the chemical reaction that occurs during discharging.

**Table 2:** Discharge performances of cathodes using Fe foam and HT-500.

**Type**	**Thickness** **(mm)**	**Foam weight** **(g)**	**Total electrode weight** **(g)**	**Discharge capacity** **(A s/g)**
Using Fe foam	1.6	1.209	4.739	404.04
Using HT-500	1.6	2.183	6.118	538.38

**Figure 8:**
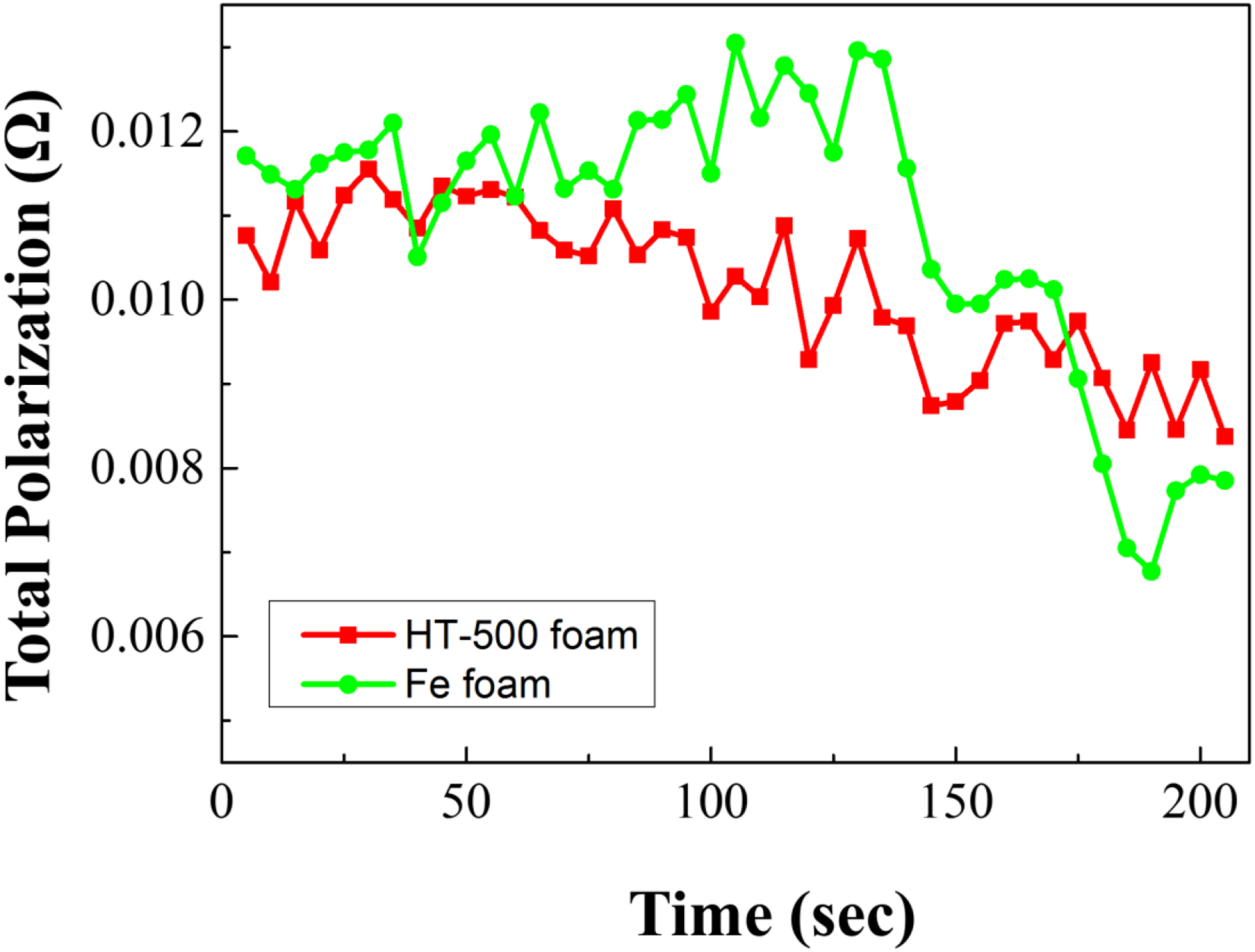
Total polarization graphs of cathodes using Fe and HT-500 foams.

Based on the discharge evaluation shown in Figure 8, the total polarization of the single battery with a cathode frame was calculated using the formula reported by Fujiwara et al. (2011). The total polarization was calculated using the following equation:

(1) $$\begin{array}{l} Rt=\frac{V_{OC}-V_{CC}}{I} \end{array}$$

where Rt: total polarization, Ω; V_oc_: open circuit voltage, V; V_cc_: close circuit voltage, V; and I: discharge current, A.

During discharge, the total polarization of the Fe foam cell is higher than that of the HT-500 cell. Furthermore, a strong internal resistance was observed during the reaction between 100 and 150 s. These results are consistent with the results in Figure 7 which shows a region where the performance of the Fe foam cell rapidly decreased. These results indicate that the internal resistance increases as an intermediate product (in the X-phase and J-phase) during the thermal battery discharge is rapidly generated because of the reaction between the Fe foam and the melted electrolyte (Kam and Johnson, 1980). In addition, the total polarization appears to be lower after 150 s, which is due to the electrons generated upon the reaction of Fe and LiS_2_, a byproduct of the thermal battery reaction (Peled and Lavi, 1998; Strauss and Peled, 2000; Masset and Guidotti, 2008a). Because this occurs when the thermal battery reaction is completed, the discharge capacity is less affected (Figure S3). These results have an impact on the discharge characteristics of the battery, causing a reduction in its capacity when compared with batteries using the HT-500 foam. Table 3 shows a comparison of the discharge characteristics of the sample fabricated in this study to those fabricated through other methods. The thermal battery discharge profile depends on the pulse current. Generally, as the discharge current increases, the operation time and capacity of the battery reduce (Wesolowski and Papenguth, 2010; Ceriotti et al., 2011). These cells can be fabricated with diameters larger than those fabricated by other methods.

**Table 3:** Characteristics of the cathodes based on production method.

**Sample**	**Electrode manufacturing method**	**Theoretical capacity** **(A s/g) (Guidotti and Masset, 2006)**	**Capacity** **(A s/g)**	**Additive**	**Cut-off voltage (V)**	**Pulse load (A)**	**Battery size** **(mm)**
This study	Using foam	1,206	538.38 (at 500°C)	No	1.3	10	Diameter: 56.2 Thickness: 1.6
1	Plasma spray	1,206	1,967 (at 550°C) (Ulissi et al., 2018)	No	1.0	2	Diameter: 31.8 Thickness: 0.13
2	Tape casting	1,206	597 (at 550°C) (Kim et al., 2017)	Polymeric binder	1.5	2.2	Diameter: 30 Thickness: 0.134
3	Cold pressing	1,206	647.4 (at 550°C) (Kim et al., 2017)	LiF-LiBr-LiCl, MgO	1.5	2.2	Diameter: 30 Thickness: -

Furthermore, we compared the discharge performances of the cold-press electrode and the electrode using HT-500 (Figure S4). Typically, the thickness of the cathode electrode manufactured by the cold-press method is 0.5 mm, and that of the HT-500 cathode was also 0.5 mm. The former showed a capacity of 996.0 A s/g and the latter showed a capacity of 1251.2 A s/g. The capacity of the cathode using HT-500 foam was 1.2 times that of the cold-press electrode. The discharge characteristics of the single cell using the HT-500 foam show that the cell can be utilized to manufacture a thermal battery electrode.

## Conclusions

HT-500 foam was synthesized through a sulfidation process for use as a thermal cathode frame. The HT-500 foam reduced the contact resistance between the active material and the Fe foam because of the formation of FeS_2_ crystals, which was dependent on the thermal sulfidation temperature. The foam was used as a frame for thermal battery cathodes, and the discharge capacity of the cathode was measured. The discharge characteristics of the HT-500 and Fe foam cathodes were compared. The discharge capacity of a single cell with the HT-500 foam was 538.38 A s/g, which is 1.3 times higher than that of the single cell using the Fe foam. These results indicate that the discharge characteristics and mechanical strength of thermal battery cathodes can be improved by decreasing their interfacial resistance by coating active materials on their surfaces. Thus, HT-500 foam is a promising cathode frame for high-performance thermal batteries.

## Data Availability Statement

All datasets generated for this study are included in the article/Supplementary Material.

## Author Contributions

The concept for this study was designed by YY, H-WC, and J-HL. IK designed the experiment. IK and SW performed experiments on material selection and sulfidation temperature conditions. JK performed discharge evaluation. YY and J-HL performed raw material selection and helped in morphology analysis. H-WC and S-HK coordinates the interpretation of the electrochemical assessment. All authors helped write the manuscript.

### Conflict of Interest

The authors declare that the research was conducted in the absence of any commercial or financial relationships that could be construed as a potential conflict of interest.

## Abbreviations

SEM: scanning electron microscopy
EDX: energy dispersive X-ray spectroscopy
XRD: X-ray diffraction.

## References

[B1] Au M. (2003). Nanostructured thermal batteries with high power density. J. Power Sources 115, 360–366. 10.1016/S0378-7753(02)00627-4

[B2] Cabán-Acevedo M. Liang D. Chew K. S. DeGrave J. P. Kaiser N. S. Jin S. (2013). Synthesis, characterization, and variable range hopping transport of pyrite (FeS_2_) nanorods, nanobelts, and nanoplates. ACS Nano 7, 1731–1739. 10.1021/nn305833u 23330940

[B3] Ceriotti M. Corrà M. Orazio L. D. Doriguzzi R. Facchin D. Gună S. T. (2011). Is there light at the ends of the tunnel? Wireless sensor networks for adaptive lighting in road tunnels, in Proceedings of the 10th ACM/IEEE International Conference on Information Processing in Sensor Networks (Chicago, IL), 12–14.

[B4] Cha Y. L. Park I. H. Moon K. H. Kim D. H. Jung S. I. Yoon Y. S. (2018). Simultaneous control of phase transformation and crystal of amorphous TiO_2_ coating on MWCNT surface. J. Korean Ceram. Soc. 55, 618–624. 10.4191/kcers.2018.55.6.09

[B5] Choi Y. Cho S. Lee Y. S. (2014). Effect of the addition of carbon black and carbon nanotube to FeS_2_ cathode on the electrochemical performance of thermal battery. J. Ind. Eng. Chem. 20, 3584–3589. 10.1016/j.jiec.2013.12.052

[B6] Cooper K. R. Smith M. (2006). Electrical test methods for on-line fuel cell ohmic resistance measurement. J. Power Sources 160, 1088–1095. 10.1016/j.jpowsour.2006.02.086

[B7] Ferrer I. J. Sánchez C. (1991). Characterization of FeS_2_ thin films prepared by thermal sulfidation of flash evaporated iron. J. Appl. Phys. 70, 2641–2647. 10.1063/1.349377

[B8] Fujiwara S. Inaba M. Tasaka A. (2011). New molten salt systems for high temperature molten salt batteries: ternary and quaternary molten salt systems based on LiF–LiCl, LiF–LiBr, and LiCl–LiBr. J. Power Sources 196, 4012–4018. 10.1016/j.jpowsour.2010.12.009

[B9] Giagloglou K. Payne J. L. Crouch C. Gover R. K. B. Connor P. A. Irvine J. T. S. (2016). Zirconium trisulfide as a promising cathode material for Li primary thermal batteries. J. Electrochem. Soc. 163, A3126–A3130. 10.1149/2.1351614jes

[B10] Gomez J. Nelson R. Kalu E. E. Weatherspoon M. H. Zheng J. P. (2011). Equivalent circuit model parameters of a high-power Li-ion battery: thermal and state of charge effects. J. Power Sources 196, 4826–4831. 10.1016/j.jpowsour.2010.12.107

[B11] Guidotti R. Preston S. (2007). Electrode fabrication processes for thermal batteries, in 5th International Energy Conversion Engineering Conference and Exhibit (St. Louis, MO), 25–27.

[B12] Guidotti R. A. Masset P. (2006). Thermally activated (“thermal”) battery technology Part I: an overview. J. Power Sources 161, 1443–1449. 10.1016/j.jpowsour.2006.06.013

[B13] Guidotti R. A. Masset P. J. (2008). Thermally activated (“thermal”) battery technology Part IV: anode materials. J. Power Sources 183, 388–398. 10.1016/j.jpowsour.2008.04.090

[B14] Guidotti R. A. Reinhardt F. W. Dai J. Roth J. Reisner D. E. (2002). Characterization of plasma-sprayed pyrite/electrolyte composite cathodes for thermal batteries. J. New Mat. Electrochem. Syst. 5, 273–279. Available online at: https://www.researchgate.net/profile/Ronald_Guidotti/publication/267725303_Characterization_of_Plasma-Sprayed_PyriteElectrolyte_Composite_Cathodes_for_Thermal_Batteries/links/547784960cf2a961e483ac59.pdf

[B15] Guidotti R. A. Reinhardt F. W. Jinxiang D. Xiao T. D. Reisner D. (2000). Thermal-sprayed, thin-film pyrite cathodes for thermal batteries-discharge-rate and temperature studies in single cells, in 35th Intersociety Energy Conversion Engineering Conference and Exhibit (Las Vegas, NV), 24–28.

[B16] Ji H. Zhang L. Pettes M. T. Li H. Chen S. Shi L.. (2012). Ultrathin graphite foam: a three-dimensional conductive network for battery electrodes. Nano. Lett. 12, 2446–2451. 10.1021/nl300528p 22524299

[B17] Jin C. Fu L. Zhu J. Yang W. Li D. Zhou L. (2018). A hierarchical carbon modified nano-NiS_2_ cathode with high thermal stability for a high energy thermal battery. J. Mater. Chem. A. 6, 7123–7132. 10.1039/C8TA00346G

[B18] Jin C. Zhou L. Fu L. Zhu J. Li D. Yang W. (2017). The acceleration intermediate phase (NiS and Ni_3_S_2_) evolution by nanocrystallization in Li/NiS2 thermal batteries with high specific capacity. J. Power Sources 352, 83–89. 10.1016/j.jpowsour.2017.03.119

[B19] Kam K. W. Johnson K. E. (1980). Cycle voltammetry of Li_2_S, FeS and FeS_2_ in LiC1-KCl eutectic melt. J. Electroanal. Chem. 115, 53–64. 10.1016/S0022-0728(80)80495-5

[B20] Kang S. H. Lee J. U. Hur T. Cheong H. W. Yi J. (2016). Heat treatment effect on microstrain and electrochemical performance of nano-sized FeS_2_ cathode for thermal batteries. Int. J. Electrochem. Sci. 6, 4371–4379. 10.20964/2016.06.41

[B21] Kim I. Y. Shin S. Y. Ko J. H. Lee K. S. Woo S. P. Kim D. K. (2017). Functional Li-M (Ti, Al, Co, Ni, Mn, Fe)-O energy materials. J. Korean Ceram. Soc. 54, 9–22. 10.4191/kcers.2017.54.1.11

[B22] Ko J. Kim I. Y. Jung H. M. Cheong H. Yoon Y. S. (2017). Thin cathode for thermal batteries using a tape-casting process. Ceram. Int. 43, 5789–5793. 10.1016/j.ceramint.2017.01.126

[B23] Leviatan T. (2011). Development of thin layer electrochemical components for advanced thermal batteries, in 9th Annual International Energy Conversion Engineering Conference (IECEC) (San Diego, CA).

[B24] Liu X. Ho J. Y. L. Wong M. Kwok H. S. Liu Z. (2016). Synthesis, characterization and fabrication of ultrathin iron pyrite (FeS_2_) thin films and field-effect transistors. RSC Adv. 6, 8290–8296. 10.1039/C5RA23344E

[B25] Lucas J. M. Tuan C.-C. Lounis S. D. Britt D. K. Qiao R. Yang W. (2013). Ligand-controlled colloidal synthesis and electronic structure characterization of cubic iron pyrite (FeS_2_) nanocrystals. Chem. Mat. 25, 1615–1620. 10.1021/cm304152b

[B26] Masset P. Guidotti R. A. (2007). Thermal activated (thermal) battery technology Part II. molten salt electrolytes. J. Power Sources 164, 397–414. 10.1016/j.jpowsour.2006.10.080

[B27] Masset P. Schoeffert S. Poinso J.-Y. Poignet J.-C. (2005). LiF-LiCl-LiI vs. LiF-LiBr-KBr as molten salt electrolyte in thermal batteries. J. Electrochem. Soc. 152, A405–A410. 10.1149/1.1850861

[B28] Masset P. J. Guidotti R. A. (2008a). Thermal activated (“thermal”) battery technology Part IIIa: FeS_2_ cathode material. J. Power Sources 177, 595–609. 10.1016/j.jpowsour.2007.11.017

[B29] Masset P. J. Guidotti R. A. (2008b). Thermal activated (“thermal”) battery technology Part IIIb: sulfur and oxide based cathode materials. J. Power Sources 178, 456–466. 10.1016/j.jpowsour.2007.11.073

[B30] Miao R. Dutta B. Sahoo S. He J. Zhong W. Cetegen S. A.. (2017). Mesoporous iron sulfide for highly efficient electrocatalytic hydrogen evolution. J. Am. Chem. Soc. 139, 13604–13607. 10.1021/jacs.7b07044 28871790

[B31] Peled E. Lavi Y. (1998). Li/CPE/FeS_2_ rechargeable battery. Electrochim. Acta 43, 1593–1599 10.1016/S0013-4686(97)10059-7

[B32] Preto S. K. Tomczuk Z. von Winbush S. Roche M. F. (1983). Reactions of FeS_2_, CoS_2_, and NiS_2_ electrodes in molten LiCI-KCI electrolytes. Electrochem. Soc. 130, 264–273. 10.1149/1.2119692

[B33] Rahman M. A. Wen C. (2016). A study of the capacity fade of porous NiO/Ni foam as negative electrode for lithium-ion batteries. Ionics 22, 173–184. 10.1007/s11581-015-1542-8

[B34] Schoeffert S. (2005). Thermal batteries modeling, self-discharge and self-heating. J. Power Sources 142, 361–369. 10.1016/j.jpowsour.2004.09.038

[B35] Sinclair D. C. (1995). Characterization of electro-materials using ac impedance spectroscopy. Cerámicay Vidrio 32, 55–65.

[B36] Singh P. Guidotti R. A. Reisner D. (2004). AC impedance measurements of molten salt thermal batteries. J. Power Sources 138:323–326. 10.1016/j.jpowsour.2004.06.038

[B37] Strauss E. Peled E. (2000). Study of phase changes during 500 full cycles of Li/composite polymer electrolyte/FeS_2_ battery. Electrochim. Acta 45, 1519–1525 10.1016/S0013-4686(99)00368-0

[B38] Sun C. Li Y. Jin J. Yang J. Wen Z. (2019). ZnO nanoarray-modified nickel foam as a lithiophilic skeleton to regulate lithium deposition for lithium-metal batteries. J. Mater. Chem. A 7, 7752–7759. 10.1039/C9TA00862D

[B39] Thomas B. Cibik T. Höpfner C. Diesner K. Ehlers G. Fiechter S. (1998). Formation of secondary iron-sulphur phases during the growth of polycrystalline iron pyrite (FeS_2_) thin films by MOCVD. J. Mater. Sci. Mater. Electron. 9, 61–64. 10.1023/A:1008888801424

[B40] Ulissi U. Ito S. Hosseini S. M. Varzi A. Aihara Y. Passerini S (2018). High capacity all-solid-state lithium batteries enabled by pyrite-sulfur composites. Adv. Energy Mater. 8:1801462 10.1002/aenm.201801462

[B41] Wesolowski D. E. Papenguth H. W. (2010). Study of cell performance in long-life thermal battery design space, in 44th Power Sources Conference (Las Vegas, NV), 14–17.

[B42] Wu Z. Sun L. Wang J. (2018). Progresses on the optimal processing and properties of highly porous rare earth silicate thermal insulators. J. Korean Ceram. Soc. 55, 527–555. 10.4191/kcers.2018.55.6.12

[B43] Yang Z. Liu X. Feng X. Cui Y. Yang X. (2014). Hydrothermal synthesized micro/nano-sized pyrite usedas cathode material to improve the electrochemical performance of thermal battery. J. Appl. Electrochem. 44, 1075–1080. 10.1007/s10800-014-0724-9

