# Supplementary material for: Binder-Free Cathode for Thermal Batteries Fabricated Using FeS2 Treated Metal Foam
Source: Front Chem. 2020 Jan 10;7:904. doi: 10.3389/fchem.2019.00904 (PMC6966698; doi:10.3389/fchem.2019.00904)
Supplement: Supplementary file 1 [file Data_Sheet_1.docx]

Supporting Information

Binder-Free Cathode for Thermal Batteries Fabricated Using FeS_2_ Treated Metal Foam

**In Yea Kim^1^, Sung Pil Woo^2^, Jaehwan Ko^1^, Seung-Ho Kang^3^, Young Soo Yoon^1^, Hae-Won Cheong^3*^, and Jae-Hong Lim^1*^**

^1^Department of Materials Science and Engineering, Gachon University, 1342 Seongnamdearo, Republic of Korea

^2^Department of Materials Science and Engineering, Yonsei University, 50 Yonseiro, Republic of Korea

^3^Agency for Defense Development, P.O. Box 35-4, Daejeon 34186, Republic of Korea

Running title: Binder-free cathode electrode for thermal batteries

***Correspondence:**
E-mail: limjh@gachon.ac.kr (J.-H. Lim),

[hwcheong@add.re.kr](mailto:hwcheong@add.re.kr) (H.-W Cheong).

**Keywords: Thermal battery, FeS_2_ foam, Metal foam, Thermal sulfidation, Cathode frame.**

**
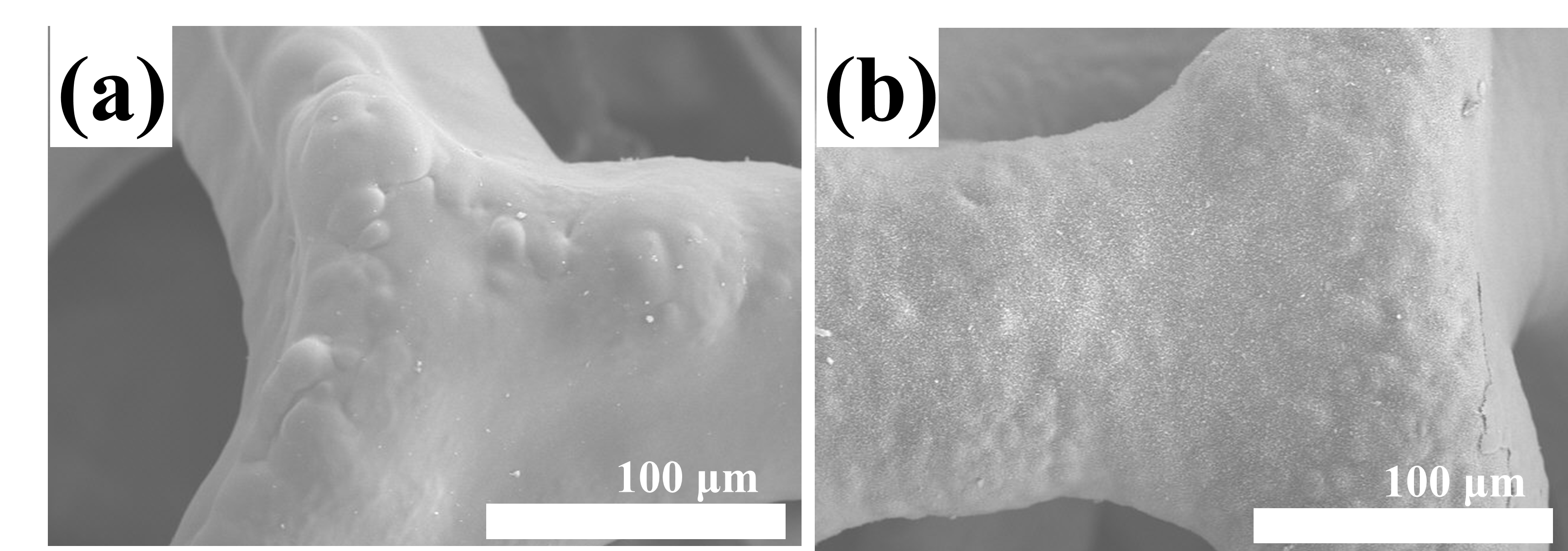
**

Figure S1. SEM images of (a) HT-400 and (b) HT-500 foams.

**
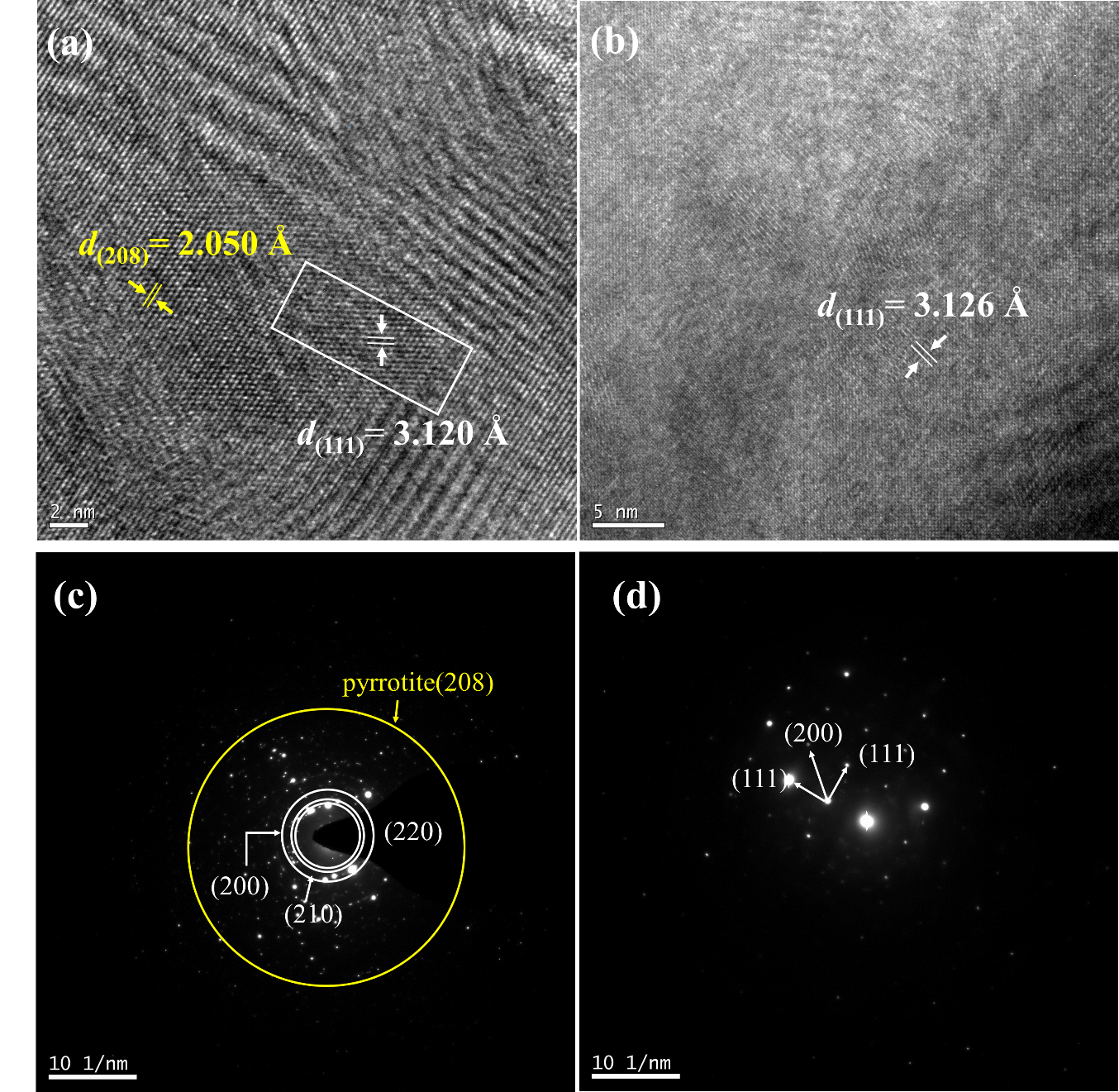
**

Figure S2. HRTEM images of (a) HT-400 and (b) HT-500 foams. SAED patterns of (c) HT-400 and (d) HT-500 foams.

Figure S3. Discharge graphs according to the working time of cathodes using Fe and HT-500 foams.


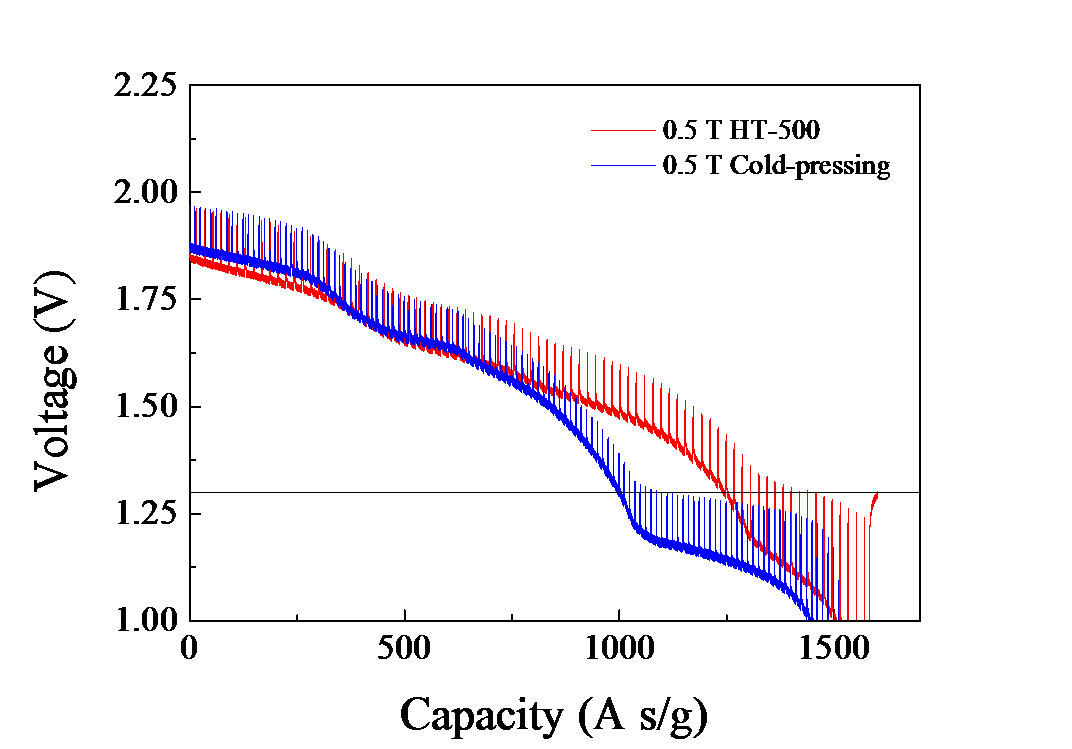


Figure S4. Discharge graphs of cathodes using cold-press and HT-500 foams.
